# Parameter Identification in a Tuberculosis Model for Cameroon

**DOI:** 10.1371/journal.pone.0120607

**Published:** 2015-04-13

**Authors:** Dany Pascal Moualeu-Ngangue, Susanna Röblitz, Rainald Ehrig, Peter Deuflhard

**Affiliations:** 1 Institute of Horticultural Production Systems, Leibniz Universität Hannover, Hannover, Germany; 2 Department of Numerical Mathematics, Zuse Institute Berlin (ZIB), Berlin, Germany; 3 Beijing Center for Scientific and Engineering Computing, Beijing University of Technology, Beijing, China; Fundació Institut d’Investigació en Ciències de la Salut Germans Trias i Pujol. Universitat Autònoma de Barcelona. CIBERES, SPAIN

## Abstract

A deterministic model of tuberculosis in Cameroon is designed and analyzed with respect to its transmission dynamics. The model includes lack of access to treatment and weak diagnosis capacity as well as both frequency- and density-dependent transmissions. It is shown that the model is mathematically well-posed and epidemiologically reasonable. Solutions are non-negative and bounded whenever the initial values are non-negative. A sensitivity analysis of model parameters is performed and the most sensitive ones are identified by means of a state-of-the-art Gauss-Newton method. In particular, parameters representing the proportion of individuals having access to medical facilities are seen to have a large impact on the dynamics of the disease. The model predicts that a gradual increase of these parameters could significantly reduce the disease burden on the population within the next 15 years.

## Introduction

Tuberculosis (TB) is a preventable and curable disease caused by *Mycobacterium tuberculosis* (Mtb) that most often affects the lungs. To date, TB claims the second largest number of fatalities due to a single infectious agent right after Human Immunodeficiency Virus and Acquired Immune Deficiency Syndrome (HIV/AIDS) [1]. Young adults in their most productive years are especially affected. Worldwide, 8.8 million people were infected in 2010. Sub-Saharan Africa carries the greatest burden with over 260 new cases per 100,000 population in 2011 [1]. Structurally speaking, Mtb’s infection can remain latent, become active, or progress from latent TB to active TB either by endogenous re-activation and/or exogenous re-infection. Active TB is often acquired through co-infection of Mtb with other diseases (such as diabetes and HIV/AIDS) or abuse of alcohol and tobacco.

Mathematical analysis of disease transmission models can significantly help to understand the underlying mechanisms of these processes and to design potential therapies [2, 3]. The earliest mathematical models describing TB dynamics were constructed in the 1960’s by the statistician H. T. Waaler [4]. His models focus on prediction and control strategies using simulation approaches. However, he considered exponential population dynamics in the absence of TB, which is certainly not realistic. Further simulation models have been suggested by Waaler [5], Revelle and coworkers [6], and Ferebee [7]. Revelle [6] first introduced a TB dynamics model consisting of systems of nonlinear ordinary differential equations (ODEs), which he used to study a minimal cost strategy against TB. Later, Blower and colleagues [8–10] discussed the persistence condition of TB inside the population and determined the characteristic reproduction ratio ℛ_0_, the average number of new infectious cases caused by a single infectious case in a fully susceptible population over the course of the entire infectious period. A sensitivity analysis of ℛ_0_ has been performed by several authors [8]. However, the sensitivity analysis of parameters with respect to ℛ_0_ alone does not fully exhibit the impact of these parameters on the global time dependent behavior of the system, especially in the presence of backward bifurcation. From their model, Blower and colleagues derived that 1 < ℛ_0_ < 9. Moreover, the infection rate, the probability of fast progression, the re-activation rate, and the TB related death rate appeared to be the most important parameters. Chavez and colleagues [11] developed a mathematical analysis of a TB model without fast progression. Since then, most publications included sophisticated mathematical theories to study the dynamics of tuberculosis, such as center manifold theory and Lyapunov functions [12–16].

In the reality of developing countries the true challenge of TB control is the high level of undiagnosed infectious cases as well as lost sight cases compared to diagnosed infectious cases. In the following, the term “undiagnosed infectious population” means those people that have not yet been to a hospital for diagnosis or have not been detected, but have a pulmonary TB [17, 18]; the term “lost sight population” includes those people that have been diagnosed as having active TB and begun their treatment, but quit before the end. Compared to existing results in [11–14, 16, 19–23] (and references therein), the model presented here considers, in addition to undiagnosed infectious and lost sight population, the aspects of exogenous re-infections, disease relapse as well as primary active TB cases, natural recovery and traditional medicine or self-medication (practiced in Sub-Saharan Africa). Moreover, our model takes undiagnosed population, lost sight population, and exogenous re-infections as important factors of TB transmission in Sub-Saharan Africa into account. As a consequence, we divide the infective class into the following three subgroups: i) diagnosed infectious population, ii) undiagnosed infectious population and iii) lost sight population. According to the National Committee for the Fight against TB in Cameroon (NCFT) [24], about 8% of diagnosed infectious people who begin a therapy, never return to the hospital for the rest of sputum examinations and treatment, and then become part of the lost sight population. The design of the model is accompanied by a qualitative analysis to gain insight into the transmission dynamics of TB.

The main difficulty in model construction is the large number of unknown model parameters. Usually, the goal is to determine parameter values that minimize the difference between experimental measurement values and model predictions in a least-squares sense. Often, however, there are not enough data available to determine all parameter values. In this case, the least-squares problems are rank-deficient, which means that there exists a continuum of possible solutions all of which give nearly the same “nice fits”. Standard optimization methods typically ignore this detail, failing to take the structure of the underlying inverse problem into account. These algorithms tend to simply return a “solution”, but do not consider the question of the identifiability of the parameters or the uniqueness of the solutions. This can yield misleading results, especially when parameters are co-regulated. In view of this situation, we solve such nonlinear least-squares problems by an error-oriented Gauss-Newton method [25], which monitors the numerical rank of the Jacobian matrix and converges locally, for a well-defined class of statistically “adequate” problems, to a solution that is unique within the subspace of identifiable parameters. As usual, special attention must be paid to initial guesses for the parameter values to be estimated. Since the algorithm converges to a local optimum, these values should reflect good approximations to the “true” values. In fact, this methodology has not been applied to the modelling of TB dynamics before and represents an important motivation for writing this paper. Alternatively, one might think of developing simpler models with less parameters. Such models, however, are prone to lose their predictive ability, if important mechanisms of disease transmission are missing. On the contrary, larger models can help in determining the importance of assumed mechanisms for the observed effects.

The paper is organized as follows. At the beginning, we derive the model equations and briefly review the mathematical algorithm used for sensitivity analysis and parameter identification. Then we show that the model is mathematically well-posed and epidemiologically reasonable. We present the values of model parameters and discuss the results of the sensitivity analysis. Finally, by means of a controlled dynamical system we simulate an increasing access to TB treatment and discuss the effect of this strategy. Even though the used data originate from Cameroon, the class of TB epidemiological models presented here can be extended to many classes of infective individuals and data for further African countries.

## Methods

This section presents our newly suggested mathematical model and briefly reviews the mathematical algorithms for sensitivity analysis and parameter identification.

### Suggested epidemiological model

Based on the available data, a finite total population at time *t* denoted by *N*(*t*) is considered and sub-divided into the following mutually exclusive sub-populations (“compartments”):

S susceptible: healthy people not yet exposed to TB
E latently infected: exposed to TB but not infectious
I diagnosed infectious: have active TB confirmed after a sputum examination in a hospital
J undiagnosed infectious: have not yet been to a hospital for diagnosis but are active for confirmation by a sputum examination
L lost sight: people who have been diagnosed as having active TB, begun their treatment and quitted before the end
R recovered: people cured after treatment in the hospital

With these definitions, we are now ready to derive the details of our compartment model.

In Africa, reliable TB tests [26] are often missing or too expensive. Hence, TB diagnosis based on a single sputum examination can often only be classified as “probable” or “presumed”, and cannot detect cases of less infectious forms of TB [27].

Transmission of Mtb occurs due to sufficient contacts among susceptible people and infectious TB cases. Diagnosed infectious people are in most cases hospitalized for at least two months or are advised to lessen their infectiousness in their neighborhood. Their distribution in the population is not necessarily homogeneous. People from class *L* (lost sight) are first diagnosed before quitting the treatment. Even though they are more likely to transmit the disease than diagnosed cases who continue their treatment, since they are aware of their infectiousness they are less likely to transmit TB than undiagnosed cases. We consider a density dependent force of infection for both diagnosed infectious people as well as lost sight infectious people, but with different contact rates for the infection [28]. Since undiagnosed infectious people remain inside the population, there is an unlimited possibility of contacts with the susceptible population [17]. Thus, susceptible individuals acquire Mtb infection from individuals with active TB (classes *I* and *J*) and lost sight people (class *L*) at a rate *ν*(*I*, *J*, *L*) given by

$$\begin{array}{c} \nu \left(I,J,L\right)=\beta_{1}\frac{I}{N}+\beta_{2}\frac{L}{N}+\beta_{3}J. \end{array}$$ (1)

Here, *β*_*i*_, *i* = 1, 2, 3, are the effective contact rates with diagnosed, lost sight and undiagnosed infectious population sufficient to transmit infection to susceptible people. The effective contact rates *β*_1, 2_ in a given population are measured in effective contacts per unit time. This may be expressed as the product of the total contact rate per unit time (*η*_*i*_) by the risk of infection (*ϕ*_*i*_) given contact between an infectious and a susceptible individual, *β*_*i*_ = *η*_*i*_ *ϕ*_*i*_.

All recruitment is into the susceptible class and occurs at an average scale Λ. The fixed survey for non-disease related death is *μ*, thus 1/*μ* is the average lifetime. Diagnosed infectious, undiagnosed infectious and lost sight population have additional constant death rates due to the disease, defined by *d*_1_, *d*_2_ and *d*_3_, respectively.

A proportion *p* of the latently-infected individuals develop fast active TB and the remainder (1−*p*) develop latent TB and enter the latent class *E*. Among latently-infected individuals developing active TB, a fraction *f* is assumed to undergo a fast progression directly to the diagnosed infectious class *I*, while the remainder (1−*f*) enters the undiagnosed infectious class *J*. We set

$$\begin{array}{c} p_{1}=pf\;\text{and}\;p_{2}=p\left(1-f\right). \end{array}$$

Once latently infected with Mtb, an individual will remain so for life, unless reactivation occurs. Latently infected individuals are assumed to acquire some immunity as a result of infection, which reduces the risk of subsequent infection but does not fully prevent it.

Due to endogenous reactivation, a fraction 1−*r*_1_ of latently infected individuals who did not receive effective chemoprophylaxis become infectious with a constant rate *k*. They reinfect after effective contact with individuals in the active TB classes or lost sight class at a rate

$$\begin{array}{c} \lambda_{e}=\sigma_{1}\nu \left(I,J,L\right), \end{array}$$

where *σ*_1_ is the factor reducing the risk of infection as a result of acquiring immunity for latently infected individuals. Among latently infected individuals who become infectious, the fraction *h* is diagnosed and treated under the “Stop TB” program [29], while the remaining fraction 1−*h* is not diagnosed and becomes undiagnosed infectious. We assume that, after some time suffering from TB, some undiagnosed infectious people decide to go to hospital with a rate *θ*. Also, we assume that among diagnosed infectious people who begin their treatment therapy, a fraction *r*_2_ of *I* take the full dose and make all the sputum examinations and will be declared cured from the disease. Some diagnosed infectious people who do not finish their dose of drugs and sputum examinations or whose treatment was unsuccessful, will not return to the hospital for the rest of sputum examinations and check-up. They will enter the class *L* of lost sight population at a constant rate *α*. Lost sight population can return to the hospital at a constant rate *δ*.

**Fig 1:**
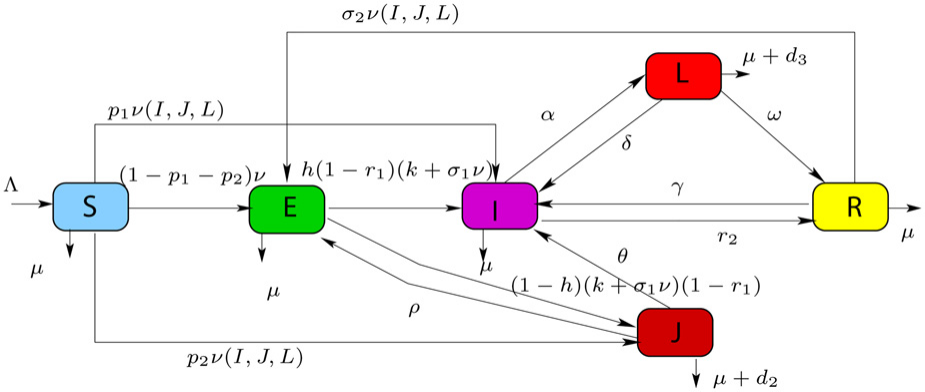
Compartment model for the transmission dynamics of tuberculosis.

As suggested by Murray et al. [30], recovered individuals can only have partial immunity. Hence, they can undergo a TB reactivation or relapse with a constant rate *γ*. The remaining people can be reinfected (exogenously) after an effective contact with individuals in the active TB classes and lost sight class at a rate

$$\begin{array}{c} \lambda_{r}=\sigma_{2}\nu \left(I,J,L\right), \end{array}$$

where *σ*_2_ is the factor reducing the risk of infection as a result of acquiring partial immunity for recovered individuals. Due to their own immunity, traditional medicine, natural recovery and drugs bought in the street (practiced in sub-Saharan Africa), a fraction of lost sight and undiagnosed infectious population can spontaneously recover at constant rates *ρ* and *ω* and enter the latent class *E* and recovery class *R*, respectively. The whole compartment model is schematically presented in a flow diagram in Fig. 1. This diagram gives rise to the following mathematical model of ordinary differential equations:

$$\begin{array}{c} \left\{\begin{array}{ccc} \dot{S} & = & \Lambda -\nu (I,J,L)S-\mu S,\\ \dot{E} & = & \left(1-p_{1}-p_{2}\right)\nu \left(I,J,L\right)S+\rho J+\sigma_{2}\nu \left(I,J,L\right)R\\ & & -\sigma_{1}\left(1-r_{1}\right)\nu \left(I,J,L\right)E-A_{1}E,\\ \dot{I} & = & p_{1}\nu \left(I,J,L\right)S+\delta L+\theta J+\gamma R+h\left(1-r_{1}\right)\left(k+\sigma_{1}\nu \left(I,J,L\right)\right)E\\ & & -A_{2}I,\\ \dot{J} & = & p_{2}\nu \left(I,J,L\right)S+\left(1-h\right)\left(1-r_{1}\right)\left(k+\sigma_{1}\nu \left(I,J,L\right)\right)E-A_{3}J,\\ \dot{L} & = & \alpha I-A_{4}L,\\ \dot{R} & = & r_{2}I+\omega L-\sigma_{2}\nu \left(I,J,L\right)R-A_{5}R, \end{array}\right. \end{array}$$ (2)

where

$$\begin{array}{c} \begin{array}{c} A_{1}=\mu +k\left(1-r_{1}\right),\;A_{2}=\mu +d_{1}+r_{2}+\alpha ,\\ A_{3}=\mu +d_{2}+\theta +\rho ,\;A_{4}=\mu +d_{3}+\delta +\omega \;\text{and}\;A_{5}=\gamma +\mu . \end{array} \end{array}$$

### Sensitivity analysis and parameter identification

In order to be able to assess targeted public health education strategies and chemoprophylaxis against TB spread in a population, we want to test the suitability of the model by fitting it to data from Cameroon [27]. For this purpose, we briefly describe the mathematical techniques that we use for parameter identification. In mathematical short-hand notation, the system of differential Equations (2) can be formally written as

$$\begin{array}{c} \left\{\begin{array}{ccc} \frac{d}{dt}y\left(t,p\right) & = & f(t,y,p),\;t\geq 0,\\ y(0,p) & = & y_{0}, \end{array}\right. \end{array}$$ (3)

where **p** is the vector of parameters, and the right-hand side *f* depends on both the states, *y* ∈ ℝ^*n*^, and the parameter vector, **p** ∈ ℝ^*q*^. The initial condition vector, *y*_0_, has the same dimension as the state vector *y*. The TB model (2) can be written in the form of Equation (3), where

$$\begin{array}{c} y\left(t,p\right)=\left(S\left(t,p\right),E\left(t,p\right),I\left(t,p\right),J\left(t,p\right),L\left(t,p\right),R\left(t,p\right)\right)\in {\mathbb{R}}^{6} \end{array}$$

and

$$\begin{array}{c} p=\left(\Lambda ,\beta ,\cdots ,\mu \right)\in {\mathbb{R}}^{22}. \end{array}$$

Assume there are given *m* experimental measurement time-point *τ*_1_, ⋯, *τ*_*m*_, and corresponding data values *z*_*j*_ ∈ ℝ^*n*^, *j* = 1, ⋯, *m* associated with corresponding measurement tolerances *δz*_*j*_ ∈ ℝ^*n*^. For ease of presentation, these tolerances are assumed to be positive, but the algorithm to be described is also able to tackle strictly zero tolerances (that indicate equality constraints); details are omitted here, but can be found, e.g., in the book [25]. Parameter identification consists of solving the least-squares minimization problem

$$\begin{array}{c} g\left(p\right)=\frac{1}{m}\sum_{j=1}^{m}∥D_{j}^{-1}\cdot \left(y\left(\tau_{j},p\right)-z_{j}\right){∥}_{2}^{2}⟶\min_{p} \end{array}$$ (4)

with diagonal weighting

$$\begin{array}{c} D_{j}≔diag\left({\left(\delta z_{j}\right)}_{1},\cdots ,{\left(\delta z_{j}\right)}_{n}\right)\in {ℳ}_{n}\left(\mathbb{R}\right),\;j=1,\cdots ,m. \end{array}$$ (5)

That means we want to minimize the relative deviation of model and data at the measurement time points *τ*_*j*_. Again in short-hand notation, the minimization problem (4) can be written as

$$\begin{array}{c} g\left(p\right)≔F{\left(p\right)}^{T}\cdot F\left(p\right)\to \min_{p}, \end{array}$$ (6)

where *F*(**p**) = (*F*_1_(**p**), …,*F*_*m*_(**p**)) is a vector of length *N* = *m*⋅*n* with entries defined by

$$\begin{array}{c} F\left(p\right)=\left[\begin{array}{c} D_{1}^{-1}\cdot \left(y\left(\tau_{1},p\right)-z_{1}\right)\\ \vdots \\ D_{m}^{-1}\cdot \left(y\left(\tau_{m},p\right)-z_{m}\right) \end{array}\right]. \end{array}$$ (7)

*F*:ℝ^*q*^ → ℝ^*N*^ is a non-linear mapping and structured as a stacked vector. If not all components of a measurement *z*_*j*_ are given, the number *N* is accordingly smaller, *N* < *nm*. The above problem (6), which is highly nonlinear in **p**, can be solved by affine covariant Gauss-Newton iteration, see [25], where each iteration step *k* requires the solution of a linear least-squares problem,

$$\begin{array}{c} ∥J\left(p^{k}\right)\cdot \Delta p^{k}+F\left(p^{k}\right){∥}_{2}\to \min_{p^{k}}, \end{array}$$ (8)

$$\begin{array}{c} p^{k+1}=p^{k}+\Delta p^{k} \end{array}$$

where *J*(**p** ^*k*^) = *F*′(**p** ^*k*^) ∈ ℝ^*N*×*q*^ denotes the Jacobian matrix or, equivalently, the sensitivity matrix. Its elements are the *sensitivities*

$$\begin{array}{c} s_{ij}\left(t\right)=\frac{\partial y_{i}\left(t\right)}{\partial p_{j}} \end{array}$$ (9)

computed at all data time-points. These entries represent the sensitivity of the solution *y* with respect to the parameters **p** at the time points of measurements. In model (2), values of model parameters and population classes vary over orders of magnitude. To achieve comparability, the sensitivity values have to be normalised by the absolute values of species and parameters to obtain *scaled sensitivities*

$$\begin{array}{c} s_{ij}\left(t\right)=\frac{\partial y_{i}\left(t\right)}{\partial p_{j}}\cdot \frac{|p_{\text{scal}}|}{|y_{\text{scal}}|}, \end{array}$$ (10)

whereby *p*_scal_ and *y*_scal_ are scaling values specified by the user.

An analysis of the scaled matrix *J*(**p**) gives some hints whether the current combination of model and data will allow an identification of a given parameter. Parameters with very small sensitivity have nearly no influence on the solution at the measurement time-points and therefore cannot be estimated. In this case the entries of the corresponding column in *J*(**p**) are almost zero. Furthermore, some of the parameters might be linearly dependent, which leads to nearly identical columns in *J*(**p**). In both cases the matrix *J*(**p**) will be singular or, from a numerical point of view, “nearly” singular. In order to reveal such properties, the linear least squares problem (8) is solved by QR factorization with column pivoting [31]. By a suitable permutation of the columns of the matrix *J*(**p**), the diagonal elements of the upper triangular matrix *R* can be ordered in the form

$$\begin{array}{c} |r_{11}\left|\geq \right|r_{22}\left|\geq \cdots \geq \right|r_{qq}|\geq 0\;. \end{array}$$

As a measure of the term “nearly singular”, the sub-condition of parameter **p**_*j*_ is defined by

$$\begin{array}{c} sc_{j}=\frac{|r_{11}|}{|r_{jj}|}. \end{array}$$ (11)

Thus, the permutation of matrix columns corresponds to a new ordering of parameters according to increasing sub-condition. The sub-condition indicates whether a parameter can be estimated from the given data or not. Only those parameters can be estimated for which

$$\begin{array}{c} sc_{j}\leq 1/\varepsilon , \end{array}$$ (12)

where *ɛ* is the relative precision of the Jacobian *J*(**p**) [32]. Herein, the Jacobian has been computed with a finite difference scheme, resulting in a precision of $\sqrt{\epsilon_{\text{mach}}}$, whereby *ϵ*_mach_ = 2^−52^ is the machine precision, i.e. the floating-point relative accuracy for double precision. Thus we set *ɛ* = 10^−7^ throughout the numerical computations in this paper.

The iterative scheme (8) can be generalized to the so-called *global* Gauss-Newton method by introducing damping factors *λ*_*k*_,

$$\begin{array}{c} p^{k+1}=p^{k}+\lambda_{k}\Delta p^{k};\;0<\lambda_{k}\leq 1. \end{array}$$

The step length *λ*_*k*_ is computed successively in each iteration by an adaptive trust-region method [25]. This method for solving a non-linear least squares problem is implemented in the software code NLSCON [25] and part of the software package BioPARKIN [33]. Here, a Matlab-based version of this software package, named POEM 2.0, which is especially adapted to parameter identification in ordinary differential equation models, has been used.

As the data set for parameter identification, we used figures for diagnosed infectious and total population in Cameroon over the period 1994–2010 as published by WHO [1]. The data are listed in Table 1.

**Table 1:** Numbers for diagnosed infectious (*I*) and total population (*N*) in Cameroon over the period 1994–2010.

year	I	N
1994	3092	13240337
1995	3292	13940337
1996	3049	14287475
1997	3952	14631908
1998	5022	14976200
1999	7660	15324051
2000	5251	15678269
2001	11307	16039737
2002	11057	16408085
2003	15964	16783366
2004	17655	17165267
2005	21499	17553589
2006	23483	17948395
2007	24062	18350022
2008	24622	18758778
2009	24662	19175028
2010	24073	19598889

Data published by WHO [27].

Overall, parameter identification is an iterative process because the sub-conditions depend on the current parameter values. At the beginning, all parameter values are fixed to their initial guesses and those parameter values are estimated that have the lowest sub-condition. These parameters are then fixed to their estimated values. Afterwards, parameter estimation is performed with a restricted list of previously non-identifiable parameters. After this phase, the estimation process is repeated with the full set of estimated parameters to check, whether the new values of previously non-identifiable parameters affect values of the identifiable ones. This process is repeated until convergence in the parameter values is achieved.

## Results and Discussion

In the following, we show that the model is mathematically well-posed and epidemiologically reasonable [34]. Furthermore, we present the values of model parameters and discuss the results of the sensitivity analysis. In addition, we analyze the effect of increasing access to TB treatment as a result of improved infrastructures and education.

### Basic properties of the model

Since model (2) monitors a human population, all its associated parameters and state variables should be non-negative and bounded for all *t* ≥ 0.

#### Positivity of the solution

The following result shows that state variables are non-negative and dissipative.

**Lemma 1** Let the initial values be *S*(0) > 0, *E*(0) ≥ 0, *I*(0) ≥ 0, *J*(0) ≥ 0, *L*(0) ≥ 0 and *R*(0) ≥ 0. Then, solutions (*S*, *E*, *I*, *J*, *L*, *R*) of model system (2) are non-negative for all *t* > 0. Furthermore,

$$\begin{array}{c} \underset{t⟶\infty }{lim sup}N\left(t\right)\leq \frac{\Lambda }{\mu }, \end{array}$$

with *N*(*t*) = *S*(*t*)+*E*(*t*)+*I*(*t*)+*J*(*t*)+*L*(*t*)+*R*(*t*).

The proof of this Lemma follows the ideas from [35, 36]. The following steps establish the invariance of the set

$$\begin{array}{c} \Omega_{\rho }=\left\{\left(S,E,I,J,L,R\right)\in {\mathbb{R}}_{+}^{6},\;\;\;N\left(t\right)\leq \frac{\Lambda }{\mu }+\rho \right\},\;\;\rho >0, \end{array}$$ (13)

i.e. solutions remain in Ω_*ρ*_ for all *t* ≥ 0. This implies that the trajectories of model system (2) are bounded. On one hand, integrating the differential inequality $\dot{N}\leq \Lambda -\mu N$ yields

$$\begin{array}{c} N\left(t\right)\leq N\left(0\right)e^{-\mu t}+\frac{\Lambda }{\mu }\left(1-e^{-\mu t}\right). \end{array}$$

In particular $N(t)\leq \frac{\Lambda }{\mu }$ if $N(0)\leq \frac{\Lambda }{\mu }$. On the other hand, if $N(0)\geq \frac{\Lambda }{\mu }$, then Λ−*μN*(0) ≤ 0, and

$$\begin{array}{c} \dot{N}\left(0\right)\leq \Lambda -\mu N\left(0\right)\leq 0, \end{array}$$

i.e. the total population *N*(*t*) will decrease until

$$\begin{array}{c} N\left(t\right)\leq \frac{\Lambda }{\mu }. \end{array}$$

Thus, the simplex Ω_*ρ*_ is a compact forward invariant set for model system (2), and for *ρ* > 0 this set is absorbing. So, we limit our study to this simplex for *ρ* > 0. The prevalent existence, uniqueness and continuation results hold for model system (2) in Ω_*ρ*_.

#### Basic reproduction number

The global behavior of the TB model crucially depends on the basic reproduction number, i.e., the average number of secondary cases produced by a single infective individual who is introduced into an entirely susceptible population. Obviously, model system (2) has an equilibrium *Q*_0_ = (*x*_0_, 0) with *x*_0_ = Λ/*μ* when *I* = 0. This equilibrium point is the *disease-free equilibrium* (DFE). On this basis, we are now able to calculate the basic reproduction number ℛ_0_, using the next generation method developed in [37]. For that purpose, let us write system (2) in the form

$$\begin{array}{c} \left\{\begin{array}{ccc} \dot{x} & = & \varphi (x)-\nu (I,J,L)x,\\ \dot{y} & = & ℱ(x,y)+𝒱(x,y), \end{array}\right. \end{array}$$ (14)

where

$$\begin{array}{c} \varphi \left(x\right)=\Lambda -\mu \;x,\;ℱ\left(x,y\right)=\nu \left(I,J,L\right)B_{1}x,\;𝒱\left(x,y\right)=\nu \left(I,J,L\right)\left[B_{2}\left\langle e_{1}|y\right\rangle +B_{3}\left\langle e_{5}|y\right\rangle \right]+A\;y. \end{array}$$ (15)

Herein, *x* = *S* ∈ ℝ_+_ is a state representing the compartment of non transmitting individuals (susceptible), $y={\left(E,I,J,L,R\right)}^{T}\in {\mathbb{R}}_{+}^{5}$ is the vector representing the state compartment of different infected individuals, and

$$\begin{array}{c} \nu \left(I,J,L\right)=\frac{\left\langle e_{1}|y\right\rangle }{N}+\left\langle e_{2}|y\right\rangle ,\;N=x+y_{1}+y_{2}+y_{3}+y_{4}+y_{5} \end{array}$$

is the force of infection. ⟨⋅∣⋅⟩ is the usual scalar product and *A* is the constant matrix

$$\begin{array}{c} A=\left[\begin{array}{ccccc} -A_{1} & 0 & \rho & 0 & 0\\ kh(1-r_{1}) & -A_{2} & \theta & \delta & \gamma \\ k\left(1-h\right)\left(1-r_{1}\right) & 0 & -A_{3} & 0 & 0\\ 0 & \alpha & 0 & -A_{4} & 0\\ 0 & r_{2} & 0 & \omega & -A_{5} \end{array}\right], \end{array}$$

with *A*_1_, *A*_2_, *A*_3_, *A*_4_ and *A*_5_ defined as above in (2). Furthermore, we set

$$\begin{array}{c} e_{1}=(0,\beta_{1},\beta_{2},0,0)\in {\mathbb{R}}^{5},\;e_{2}=(0,0,0,\beta_{3},0)\in {\mathbb{R}}^{5},\;e_{3}=(1,0,0,0,0)\in {\mathbb{R}}^{5},\;e_{4}=(0,0,0,0,1)\in {\mathbb{R}}^{5}, \end{array}$$

$$\begin{array}{c} B_{1}={\left(1-p_{1}-p_{2},p_{1},p_{2},0,0\right)}^{T}\in {\mathbb{R}}^{5}, \end{array}$$

$$\begin{array}{c} B_{2}={\left(-\sigma_{1}(1-r_{1}),h\sigma_{1}(1-r_{1}),\sigma_{1}(1-h)(1-r_{1}),0,0\right)}^{T}\in {\mathbb{R}}^{5}, \end{array}$$

$$\begin{array}{c} B_{3}={\left(-\sigma_{2}(1-\gamma ),0,0,0,\sigma_{2}(1-\gamma )\right)}^{T}\in {\mathbb{R}}^{5}. \end{array}$$

We define the Jacobian matrices at the DFE as

$$\begin{array}{c} F=\frac{\partial ℱ}{\partial y}\left(Q_{0}\right)\;\text{and}\;V=\frac{\partial 𝒱}{\partial y}\left(Q_{0}\right). \end{array}$$

Using the same notations as in [37], the basic reproduction number is given by the spectral radius of *FV* ^−1^,

$$\begin{array}{c} {ℛ}_{0}=\rho \left(FV^{-1}\right). \end{array}$$ (16)

For model (14), one has

$$\begin{array}{c} F=B_{1}\left(e_{1}+\frac{\Lambda }{\mu }e_{2}\right)\;\;\text{and}\;\;V=-A. \end{array}$$

Then, according to [38, 39], the basic reproduction ratio is given by

$$\begin{array}{c} {ℛ}_{0}=\left\langle e_{1}+\frac{\Lambda }{\mu }e_{2}|\left(-A^{-1}\right)B_{1}\right\rangle . \end{array}$$ (17)

The following result is established from [37].

**Lemma 2**: The disease-free equilibrium *Q*_0_ of model system (2) is locally asymptotically stable whenever ℛ_0_ < 1, and unstable, if ℛ_0_ > 1.

From a biological point of view, Lemma 2 implies that TB can be eliminated from the community (when ℛ_0_ ≤ 1) if the initial size of the population is in the basin of attraction of *Q*_0_. But if ℛ_0_ > 1 the infection will be able to spread through the population. Generally, the larger the value of ℛ_0_, the harder it is to control the epidemic.

### Parameter values of the model

**Fig 2:**
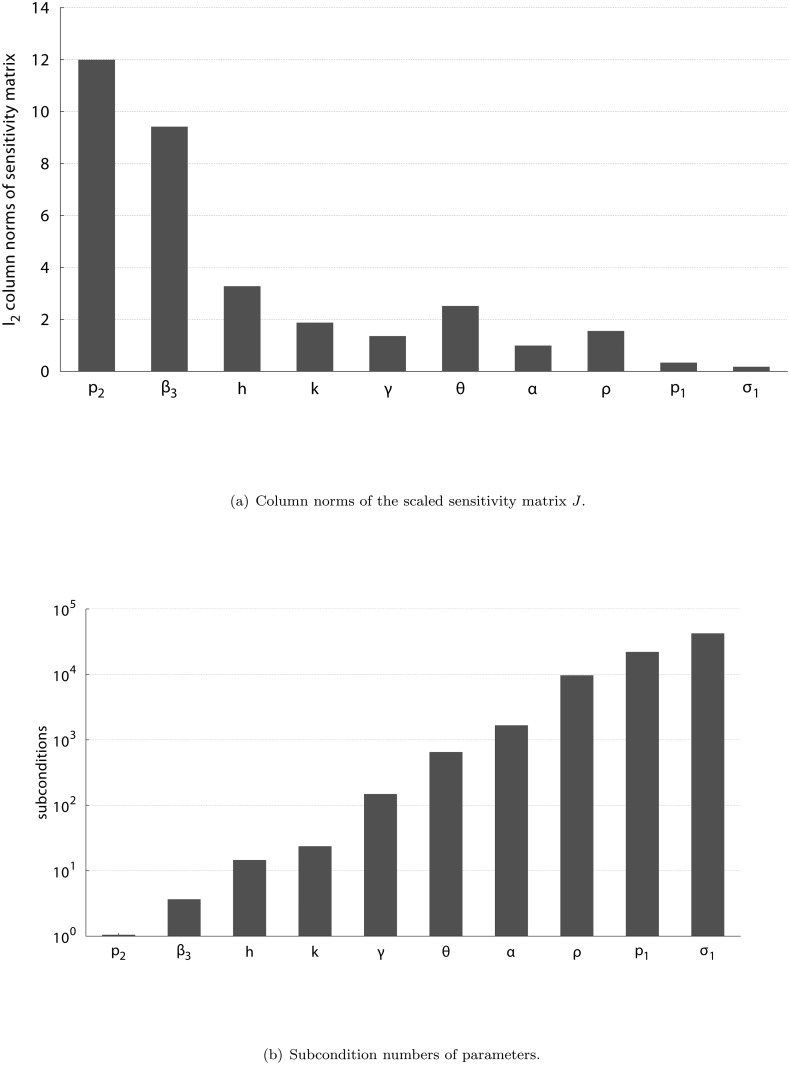
Results of the sensitivity analysis at the final iterate, i.e. with the final set of parameter values from Table 2: (a) *l*_2_ column norms of the scaled sensitivity matrix *J* and (b) subcondition numbers of parameters. In fact, all unknown parameters are identifiable for *ɛ* = 10^−7^. The magnitudes of the sensitivities alone, represented by the column norms, are an indicator for identifiability but generally do not provide information on the correct ordering of parameters.

Fig. 2 shows the column norms ‖*J*(**p**)(:, *j*)‖_2_ of the sensitivity matrix evaluated at the final iterate, i.e. for the final set of parameter values from Table 2. Obviously, the parameter with the largest column norm is *p*_2_, which means that it has the largest influence on the solution trajectory at the given measurement time-points. In fact, Fig. 2 shows that *p*_2_ is the parameter with lowest subcondition, i.e. it can be best identified from the given measurements. In contrast, parameters *σ*_1_ and *p*_1_ are the two parameters with smallest column norm and largest subcondition. Note, however, that the magnitudes of the sensitivities alone, represented by the column norms in Fig. 2, are only an indicator for identifiability but generally do not provide information on the correct ordering of parameters. Moreover, the sensitivities and subconditions change throughout the modelling process whenever parameter values are changed.

**Table 2:** Numerical values of the TB model parameters.

Parameters	Symbol	Estimate/yr	Source
Recruitment rate of susceptible	Λ	679685	Fixed, [40]
Transmission rate	*β*_1_, *β*_2_	1, 4	Fixed, [41]
Transmission rate	*β*_3_	6.33563 ⋅ 10^−06^	Estimated
Fast route to infectious class	*p*_1_	9.50082 ⋅ 10^−04^	Estimated
Fast route to undiagnosed infectious class	*p*_2_	2.38932 ⋅ 10^−02^	Estimated
Reinfection parameter of latently infected individuals	*σ*_1_	2.38390 ⋅ 10^−04^	Estimated
Reinfection parameter of recovered individuals	*σ*_2_	0.7*(*p*_1_+*p*_2_)	Fixed, [20]
Slow route to active TB	*k*	3.31390 ⋅ 10^−04^	Estimated
Natural mortality	*μ*	1/53.6	Fixed, [40]
TB mortality of diagnosed infectious	*d*_1_	0.139	Fixed, [8, 24]
TB mortality of undiagnosed infectious	*d*_2_	0.20	Fixed, [8, 24]
TB mortality of lost sight	*d*_3_	0.413	Fixed, [8]
Chemoprophylaxis of latently infected individuals	*r*_1_	0	Fixed, [24, 44]
Detection rate of active TB	*h*	0.81228	Estimated
Recovery rate of diagnosed infectious	*r*_2_	0.758821	Fixed, [24]
Recovery rate of lost sight	*ω*	0.5	Fixed
Recovery rate of undiagnosed infectious	*ρ*	0.131140	Estimated
Relapse of recovered individuals	*γ*	8.51257 ⋅ 10^−02^	Estimated
Diagnosed infectious route to the lost sight class	*α*	0.215698	Estimated
Lost sight route to the diagnosed infectious class	*δ*	0.309	Fixed, [24]
Diagnosed rate	*θ*	0.497458	Estimated

Parameters not contained in the two figures have been estimated by WHO, are well-known for Cameroon [24] or have fixed values according to the National Institute of Statistic of Cameroon (NIS) [40]. In the following, we specify all parameter values.

*The natural mortality μ*: It is postulated to be equal to the inverse of the life expectancy at birth. We fix *μ* = 1/53.6 per year according to [40].

*The recruitment Λ*: According to NIS [40], the average recruitment in the Cameroonian population during the last fifteen year is fixed to Λ = 679685 per year.

*TB mortality *d*_1_, *d*_2_ and *d*_3_ of undiagnosed infectious and lost sight population*: Per capita TB-induced mortality rate is 0.193 per year in developed countries, but could be as high as 0.45 per year in some African countries [41]. We fixed the yearly TB-induced mortality rates *d*_1_ = 0.193, *d*_2_ = 0.20 and *d*_3_ = 0.413 TB active cases according to [8, 24].

*Transmission rates *β*_*i*_, *i* = 1, 2, 3*: Blower et al. [41] estimated the contact rates *β*_*i*_ ∈ [1, 4] in the case of a frequency dependent force of infection. Here, the fixed values *β*_1_ = 1, *β*_2_ = 4 according to the data of Blower et al. have been used, and we estimated *β*_3_ = 6.33563⋅10^−06^ using POEM.

*Progression rate parameters *p*_1_, *p*_2_ and *k**: For HIV-negative TB people, Bacaer et al. [20, 42] estimated that people in a South Africa township have 11% annual risk of developing primary TB disease during five years following the first Mtb infection and a 0.03% annual risk of reactivation after five years. In Cameroon, the estimated average TB prevalence for all forms in HIV-positive is about 431 per 100,000 per year. Starting with this order of magnitude, we estimated that *p*_1_ = 9.50082⋅10^−04^, *p*_2_ = 2.38932⋅10^−02^ and *k* = 3.31390⋅10^−04^ per year. Due to the limited access to hospitals, *p*_2_ is expectedly larger than *p*_1_ and *k*.

*Factors *σ*_1_ and *σ*_2_*: Sutherland et al. [43] estimated that a previous Mtb infection reduces the risk of disease after reinfection by 63% for HIV negative males and by 80% for HIV negative females. We estimate that *σ*_1_ = 2.38390⋅10^−04^ and we use the formula from [20] to set *σ*_2_ = 0.7*(*p*_1_+*p*_2_). Running the simulation with other values for the pre-fractor, e.g. 0.2 or 0.9, leads only to small changes in the simulation result, indicating that *σ*_1_ is indeed a less sensitive parameter that is difficult to estimate from the given data.

*Detection rate *h**: According to WHO data, *h* ∈ [0.5, 0.9] per year. Using POEM, it was estimated *h* = 0.812279 per year.

*Diagnosis rate *θ**: WHO estimated *θ* ∈ [0.3, 0.6] per year. The results in Fig. 2 show that the model is highly sensitive to *θ*, which was finally estimated from the data with POEM as *θ* = 0.497358 per year.

*Proportions *r*_1_ and *r*_2_ of successful treatments*: Since the chemoprophylaxis is not practiced in Cameroon, we took *r*_1_ = 0 per year and fixed *r*_2_ = 0.758821 per year according to [24].

*Rate *α* at which diagnosed infectious people become lost sight*: It has been estimated using POEM as *α* = 0.215698 per year.

*Rate *δ* at which lost sight people return to the hospital*: According to the data of TB in Cameroon [24], we fixed *δ* = 0.309 per year.

*Natural recovery rate *ρ**: In [20], the authors estimated that the natural recovery for HIV-negative TB and HIV-positive TB cases are 0.139 and 0.24 per year, respectively. Herein, we took the average of these values as initial guess for the Gauss-Newton algorithm and estimated *ρ* = 0.131140 per year.

*Recovery rate *ω**: We fixed *ω* = 0.5 per year. A reference cannot be given for this value. A decrease (increase) of this value leads to a slight increase (decrease) in the maximum value of population class *L* but does not qualitatively affect the dynamics of the other population classes because the average population level of *L* is small.

*Relapse rate of recovered individuals*: The average relapse rate of recovered individuals is estimated with POEM as *γ* = 0.0851257 per year.

Numerical values of all parameters are summarized in Table 2. Note that some of the parameters are not specific for Cameroon, but specific for TB or valid for developing countries or sub-Saharan Africa in general. This especially applies to those parameters taken from the studies of Blower et al. [8, 41] and Nunn et al. [44]. However, since other parameter values are Cameroon-specific, the model in fact represents a study for Cameroon.

### Interpretations in terms of model simulations

In order to illustrate the theoretical results of the foregoing analysis, numerical simulations of model system (2) are carried out using a fourth order Runge-Kutta scheme in the software Matlab, version R2009. The total population of Cameroon in 1994 is given by *N* = 13,240,337 [40]. The initial values of the other variables were set as in Table 3. We deliberately decided not to fix the population numbers *N*(*t*) as equality constraints because the data do not represent true values but rather estimates.

**Table 3:** Initial values of state variables of the TB model.

Symbol	Initial value	Source	Symbol	Initial value	Source
*S*	5296135	Estimated	*E*	7937875	Estimated
*I*	3092	Fixed (WHO)	*J*	974	Estimated
*L*	251	Estimated	*R*	2010	Estimated
*N*	13240337	Fixed [40]			

**Fig 3:**
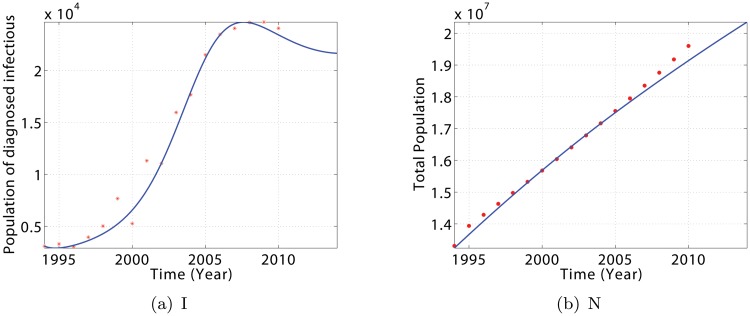
Evolution of model (2) showing the state trajectories for diagnosed infectious individuals (I) and total population (N). The dot plots represent the year-by-year trend in yearly case reports for Cameroon over the period 1994–2010. Parameter values are defined in Table 2 and initial values are presented in Table 3.

Using the data from Table 1, model (2) gives a very good fit to the Cameroonian data for the period 1994–2010 [1], as depicted in Fig. 3. The agreement between data and simulations is not perfect, but there are no evident consistent patterns in the discrepancy. Forward solutions of the deterministic model follow fairly well the observed TB patterns of incidence.

**Fig 4:**
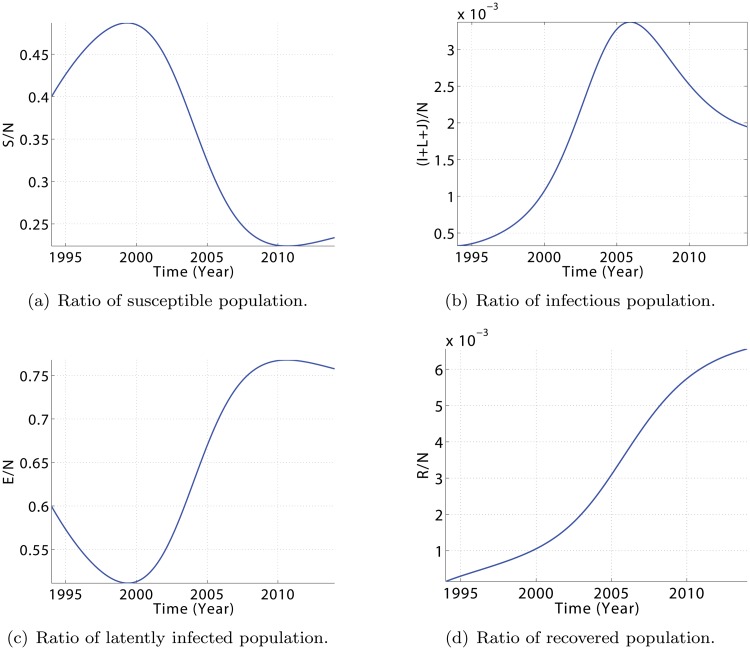
Evolution of model (2) showing the state trajectories for the relative amounts of susceptible population (*S*/*N*), latently infected population (*E*/*N*), infectious population ((*I*+*L*+*J*)/*N*), and recovered population (*R*/*N*). Parameter values are defined in Table 2 and initial values are presented in Table 3.

In addition, the obtained dynamics for the other population classes is plausible as well, see Fig. 4. To exclude the effect of a growing total population in the argumentation, numbers have been scaled with respect to *N*(*t*). Before the year 2000, the relative amount of infectious individuals, (*I*+*L*+*J*)/*N*, is small so that the contact rates to healthy individuals are low and the latently infected population (*E*) decreases. This goes along with an increase in the relative amount of healthy, susceptible individuals. (In fact, the two largest population classes, *S* and *E*, almost sum up to the total population number *N*, thus *S*/*N*+*E*/*N* ≈ 1.) Around the year 1999, however, the infectious population reaches a critical value so that the latently infected population starts increasing and the healthy, susceptible population starts decreasing. In terms of equations (compare 2), this means that $\dot{S}$ changes sign from positive to negative because now

$$\begin{array}{c} \nu \left(I,J,L\right)>\frac{\Lambda }{S}-\mu . \end{array}$$

In 2003, a new WHO strategy was applied in Cameroon, including reorganization of the National Committee on the Fight against Tuberculosis. As a consequence, the increase in the number of infectious people slowed down, reaching a maximum in 2006. With a delay of 4 years, the number of latently infected individuals also starts decreasing, hence the healthy population increases.

To summarize, with the estimated transmission parameters, the deterministic model appears to capture all the qualitative properties of the observed pattern. Hence, model (2) can be used to gain realistic insight into tuberculosis transmission dynamics at least for a limited period.

### Effects of increased access to treatment

Herein, we investigate the impact of the time variation of some specific parameters on the dynamics of model (2). For this purpose, some model parameters are considered as time dependent variables to reflect their possible change within time. However, the variation is assumed to be slow over time.

Effects of increasing the access to TB treatment as a result of improved infrastructures and education are explored by taking into account the following expressions of model parameters,

$$\begin{array}{c} \begin{array}{c} \theta \left(t\right)=\tilde{\theta }+\frac{(1-\tilde{\theta })t}{\theta_{\delta }+t},\;\delta \left(t\right)=\tilde{\delta }+\frac{(1-\tilde{\delta })t}{\delta_{\delta }+t},\\ p_{1}\left(t\right)={\tilde{p}}_{1}+\frac{{\tilde{p}}_{2}t}{p_{\delta }+t},\;p_{2}\left(t\right)={\tilde{p}}_{2}-\frac{{\tilde{p}}_{2}t}{p_{\delta }+t}. \end{array} \end{array}$$ (18)

Herein, *t* starts at 0, counting the years since the beginning of the strategy, and *θ*_*δ*_, *δ*_*δ*_ as well as *p*_*δ*_ are positive constants to be estimated. The values $\tilde{\theta },\;\tilde{\delta },\;{\tilde{p}}_{1}$ and ${\tilde{p}}_{2}$ represent the original parameter values from Table 2. The time-dependent functions have been constructed in such a way that they coincide with these values at time *t* = 0, given that *θ*_*δ*_, *δ*_*δ*_ and *p*_*δ*_ are larger than zero. Moreover, lim_*t* → ∞_ *δ*(*t*) = 1 and lim_*t* → ∞_ *θ*(*t*) = 1, i.e. the rates *δ*(*t*) and *θ*(*t*) are bounded from above by 1 as necessary. The parameters *θ*_*δ*_ and *δ*_*δ*_ determine how fast this limit is approached; the smaller their values, the faster the increase in *θ*(*t*) and *δ*(*t*), respectively. In addition, the functions *p*_1_(*t*) and *p*_2_(*t*) satisfy $p_{1}(t)+p_{2}(t)={\tilde{p}}_{1}+{\tilde{p}}_{2}$ for all *t* in order to leave the value of parameter *σ*_2_ unchanged. They have been chosen such that $\lim_{t\to \infty }p_{1}(t)={\tilde{p}}_{1}+{\tilde{p}}_{2}$ and lim_*t* → ∞_ *p*_2_(*t*) = 0. Again, the smaller *p*_*δ*_, the faster this limit is approached. Overall, the functions in Equation 18 are the simplest rational functions, i.e. with lowest polynomial order, that satisfy all the conditions discussed above.

These functions are assumed to be control functions for the dynamics of TB. Thus, model (2) becomes a non-autonomous controlled system. The goal now is to find two different sets of values for parameters *θ*_*δ*_, *δ*_*δ*_ and *p*_*δ*_ such that the following two scenarios can be achieved:

a) a reduction in the population of undiagnosed infectious (J) and lost sight (L) by 20% until 2025,
b) a reduction in the population of undiagnosed infectious (J) and lost sight (L) by 60% until 2025.

To estimate the parameter values, we generated two artificial data sets. Both data sets contain the values of J and L from the previous simulation at the adaptively chosen time-points in the time interval [1994, 2035]. However, the values in the time interval [2010, 2035] have been reduced by 20% and 60% to obtain the first and second data set, respectively. These data sets were then used in the Gauss-Newton algorithm to obtain the following estimates:

a) reduction by 20%: *p*_*δ*_ = 8.56043⋅10^7^, *θ*_*δ*_ = 81.2807, *δ*_*δ*_ = 37.2240
b) reduction by 60%: *p*_*δ*_ = 85.6660, *θ*_*δ*_ = 81.3256, *δ*_*δ*_ = 37.1301

**Fig 5:**
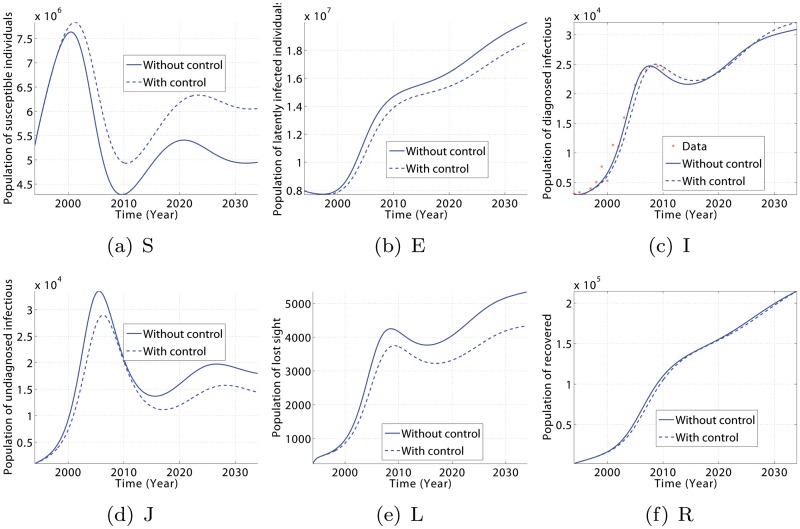
Time series of model (2) showing the impact of a slow change on parameter values*θ*, *δ*, *p*_1_ and *p*_2_ with respect to time in order to reduce the TB burden by 20% within 15 years. Solid lines present the model predictions for TB dynamics using parameter values of Table 2 and the dashed lines present the trajectories for parameters *θ*, *δ*, *p*_1_ and*p*_2_ set as in Equation (18). Parameter identification with artificial data gave*p*_*δ*_ = 8.56043⋅10^7^, *θ*_*δ*_ = 81.2807, *δ*_*δ*_ = 37.2240. All other parameters are defined as in Table 2.

**Fig 6:**
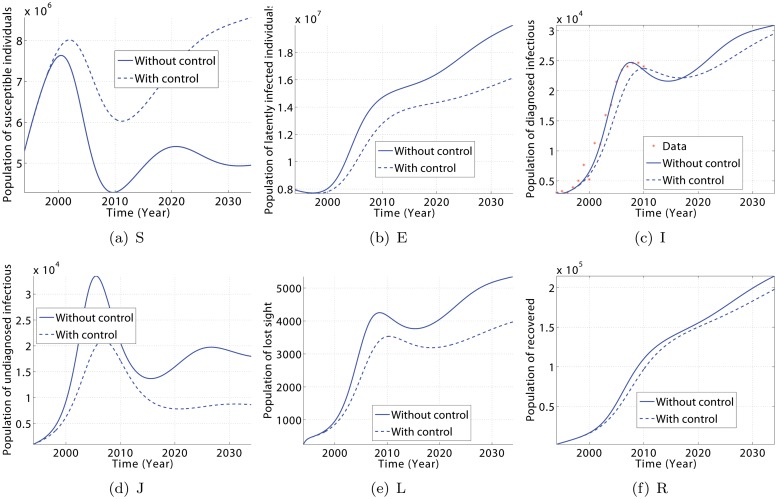
Impact of a slow change of parameter values *θ*, *δ*, *p*_1_ and *p*_2_ with respect to time in order to reduce the TB burden by 60% within 15 years. Model predictions (solid lines) for TB dynamics using parameter values of Table 2 and the estimated trajectories (dashed lines) for parameters *θ*, *δ*, *p*_1_ and *p*_2_ set as in Equation (18). Parameter identification with artificial data gave *p*_*δ*_ = 85.6660, *θ*_*δ*_ = 81.3256, *δ*_*δ*_ = 37.1301.

The simulation results are presented in Figs. 5 and 6. They show the dynamic of TB inside the population in the presence and absence of continuous effort to diagnose the population. In particular, a relatively small increase in the access to TB treatment could generally result in a decrease in the number of susceptible (S), diagnosed infectious (I), undiagnosed infectious (J), lost sight (L), and recovered (R) individuals, and an increase in the number of latently infected (E) individuals. We also observe that the number of diagnosed infectious people (I) increases at the beginning, but decreases after a few years prior to the beginning of the control strategies. Thus, TB can be reduced within 15 years if some efforts are made to increase the treatment access for rural population, and TB prevention and education for fast and immuno-compromised people.

## Conclusion

In this paper, a deterministic model for the transmission dynamics of TB in Cameroon has been presented. The objective is to determine the role of TB diagnosis, treatment, lack of information about the epidemiological status of some people, and the role of traditional medicine and natural recovery on the dynamics of TB. In contrast to other TB models in the literature, the model includes three infective classes emanating from diagnosed infectious, undiagnosed infectious, and lost sight individuals. The undiagnosed and lost sight subclasses are shown to be of particular importance in TB modeling in developing countries like sub-Saharan Africa where public health is under-developed. Model parameters have either been fixed according to data published in literature, or they have been estimated with a Gauss-Newton method using data published by WHO and the National Institute of Statistics of Cameroon (NIS). To the knowledge of the authors, it is the first time that a detailed sensitivity analysis is performed on a TB model in order to infer identifiability of unknown model parameters. In particular, parameters representing the proportion of individuals having access to medical facilities have a large impact on the dynamics of the disease. We showed that a change in these parameters over time can significantly reduce the disease burden in the population within the next 15 year. These parameters can be used to measure the success of educational and diagnosis campaigns that encourage individuals to go for TB screening. In future work, optimal control strategies could be applied to determine the optimal dynamics of these parameters in order to achieve the highest possible reduction of TB in shortest time at low costs. In addition, the model might be extended towards the inclusion of co-infection between TB and HIV, or the resistance to treatment.
